# A roundish dark spot in the upper cava vein

**DOI:** 10.1177/11297298241305726

**Published:** 2024-12-26

**Authors:** Gaetano Ferrara, Francesco Aucella, Silvio Maresca, Giovanni Ciccarese, Filippo Aucella

**Affiliations:** 1 Nephrology and Dialysis Unit, IRCCS “Casa Sollievo della Sofferenza Hospital”, San Giovanni Rotondo, 71013, Italy; 2 Interventional Radiology Unit, IRCCS “Casa Sollievo della Sofferenza Hospital”, San Giovanni Rotondo, 71013, Italy

To the Editor

A 69-year-old Caucasian woman presented to our Nephrology department with malfunction of her left-sided tunneled jugular venous hemodialysis catheter. She had been undergoing hemodialysis once a week. Her medical history includes chronic heart and renal failure, type 2 diabetes, dyslipidemia, goiter, and recurrent urinary tract infections. Initially, a temporary catheter was used due to volume overload and abnormal lab results, which was later replaced by a long-term catheter.

Four months prior, the long-term catheter was inserted in the left jugular vein using Seldinger’s technique, under B-mode ultrasound guidance.^1^ Chest X-rays post-placement confirmed correct positioning in the superior caval vein, but the catheter malfunctioned over time. Upon presentation, the patient was stable, with normal vital signs and mild inflammation on the catheter exit site in the left sub-clavicular area.^2^

Laboratory results showed elevated uric acid, BUN, and creatinine, normal hemoglobin, white blood cells, platelets, lipids, potassium, and INR, mildly elevated glucose, and slightly reduced iron levels (Table 1, lab exams).

**Table 1.** Laboratory tests.

Days	Admittance	Discharge
Blood count		
Hemoglobin (n.v.: 14–18 g/dl)	10.30	9.80
Red blood cells (n.v.: 4.7–6.10 million/µl)	3.69	3.47
White blood cells (n.v.: 4.30–10.8 thous/µl)	4.10	3.69
Platelets (n.v.: 130–400 thous/µl)	251.00	237.00
Others		
Glycemia (mg/dl, n.v.: 70–100 mg/dl)	135.00	127.00
BUN (mg/dl, n.v.: 15–38)	121.00	117.00
Creatinine (mg/dl, n.v.: 0.7–1.3 mg/dl)	5.00	4.50
Serum Na (n.v.: 135–145 mEq/l)	140.00	139.00
Serum K (n.v.: 3.5–5.0 mEq/l)	3.50	3.50
Serum chloride (v.n.: 98–107 mEq/l)	105.00	106.00
Serum uric acid	9.40	10.20
Lactic dehydrogenase (LDH, n.v.: 87–241)		
Alkaline phosphatase (n.v.: 45–117)		46.00
Serum creatine kinase (n.v.: 39–308)	88.00	
PCR HS (n.v.: <0.30)		
Homocysteine (n.v.: 4.3–11.1 mMol/l)		
D-Dimerus (n.v.: 0–500)		
Serum fibrinogen (n.v.: 150–400 mg/dl)	497.00	
Prothrombin’s time (n.v.: 70%–130%)	101	99.00
INR (n.v.: 0.8–1.2)	1.00	1.01
Gas analiter		
pH (n.v.: 7.35–7.45)	7.30	7.36
HCO3 (n.v.: 22–26 mEq/l)	19.30	20.90
BE	−4.90	−3.50
Iron plasma level (n.v.: 50–170 µg/dl)		40.00
Urine chemical/physical		
Color	Normal	
Appearance	Cloudy	
pH (n.v.: 5.0–6.5)	5.00	
Chetonics (n.v.: absent)	Absent	
Proteins (n.v.: absent)	100.00	
Specific weight	1013.00	
Blood cells (n.v.: 0 µl)	Absent	
Leukocytes	Present	
Urine culture	Negative	

A nasopharyngeal swab for SARS-CoV-2 was negative.

## Clinical investigation

**Figure 1.**
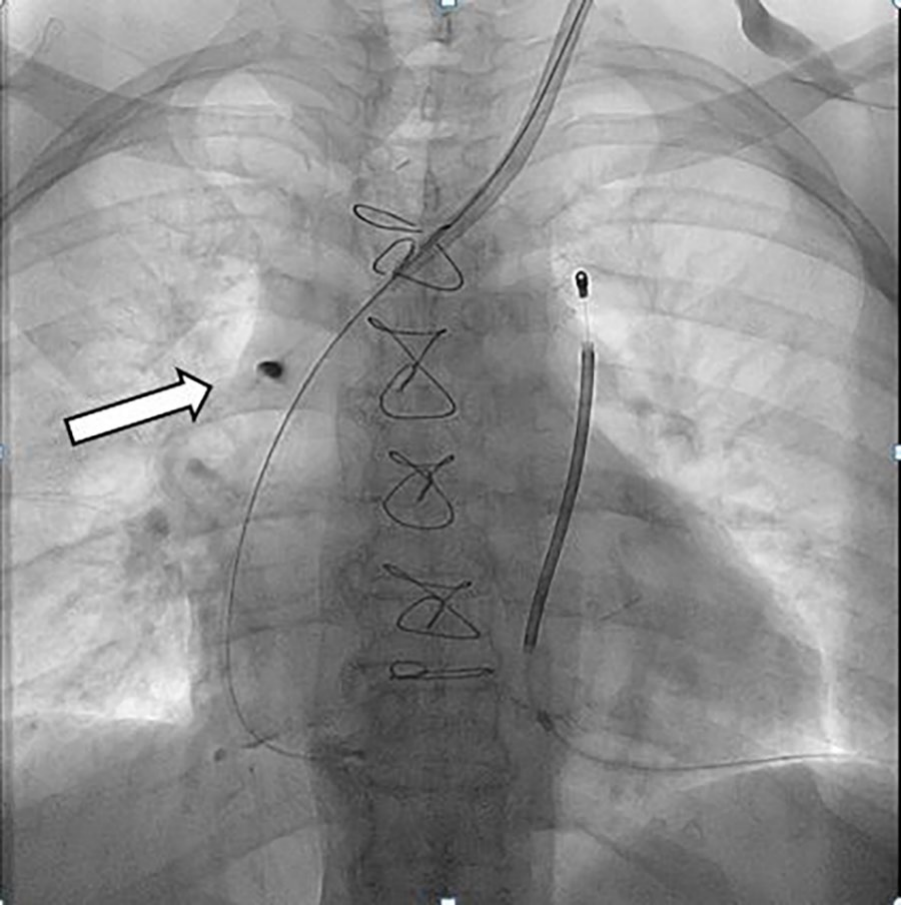
A dark roundish spot (in white arrow).

A chest X-ray revealed catheter displacement to the middle-distal superior caval vein, suggesting malfunction. This was confirmed by cavography. The catheter was replaced under fluoroscopy guidance, and a dark roundish spot was noted at the previous tip location, indicating focal intimal vascular erosion caused by prolonged contact with the vein wall^3^, (Fig. 1, a roundish dark spot in white arrow).

This erosion likely disrupted regular blood flow during hemodialysis, contributing to the catheter’s dysfunction. The exact cause of the catheter’s movement is unclear but could be linked to heart contractions, intrathoracic negative pressure, or, less likely, self-manipulation (e.g. a psychotic episode).^4^

This case highlights the potential for catheter dislocation during hemodialysis, which can compromise function and cause complications. Real-time radiological guidance is crucial for identifying and addressing catheter displacement promptly and safely.

## Conclusions

Vigilant monitoring of vascular access is essential in hemodialysis to avoid complications. Timely replacement of malfunctioning catheters under fluoroscopy is key to ensuring patient safety and continued efficacy.^5^ Catheters placed in the left jugular vein may present challenges during removal due to anatomical differences. Prolonged contact with the intimal vein wall can cause localized vascular erosion, which typically heals spontaneously and does not require additional intervention. Healthcare providers should be attentive to signs of malfunction, including poor blood flow or changes in hemodynamic stability during dialysis, as these may signal issues with catheter function.

## References

[1] Sohail MA Vachharajani TJ Anvari E . Central venous catheters for hemodialysis-the myth and the evidence. Kidney Int Rep 2021; 6(12): 2958–2968. 34901568 10.1016/j.ekir.2021.09.009 PMC8640568

[2] Gallieni M Giordano A Rossi U , et al Optimization of dialysis catheter function. J Vasc Access 2016; 17 Suppl 1: S42–S46. 10.5301/jva.5000538 26951903

[3] Kennard AL Walters GD Jiang SH , et al Interventions for treating central venous haemodialysis catheter malfunction. Cochrane Database Syst Rev 2017; 10(10): CD011953. 10.1002/14651858.CD011953.pub2 PMC6485653 29106711

[4] Wallace JA Afonso E Yu H , et al Factors that predict increased catheter tip movement in left internal jugular vein implantable venous access ports upon standing. J Vasc Access 2015; 16(3): 223–226. 25613147 10.5301/jva.5000331

[5] Zhao Y Yang L Wang Y , et al The diagnostic value of multi-detector CT angiography for catheter-related central venous stenosis in hemodialysis patients. Phlebology 2021; 36(3): 217–225. 32928071 10.1177/0268355520955090

